# Identifying an Understudied Interface: Preliminary Evaluation of the Use of Retention Ponds on Commercial Poultry Farms by Wild Waterfowl

**DOI:** 10.1155/2024/3022927

**Published:** 2024-04-03

**Authors:** Jeffery D. Sullivan, Ayla M. McDonough, Lauren M. Lescure, Diann J. Prosser

**Affiliations:** ^1^U.S. Geological Survey, Eastern Ecological Science Center, 12100 Beech Forest Road, Laurel, MD 20708, USA; ^2^Akima Systems Engineering, Contractor for U.S. Geological Survey, Eastern Ecological Science Center, 12100 Beech Forest Road, Laurel, MD 20708, USA; ^3^Student Services Contractor for U.S. Geological Survey, Eastern Ecological Science Center, 12100 Beech Forest Road, Laurel, MD 20708, USA

## Abstract

While the recent incursion of highly pathogenic avian influenza into North America has resulted in notable losses to the commercial poultry industry, the mechanism by which virus enters commercial poultry houses is still not understood. One theorized mechanism is that waterfowl shed virus into the environment surrounding poultry farms, such as into retention ponds, and is then transmitted into poultry houses via bridge species. Little is known about if and when wild waterfowl use these retention ponds, leading to uncertainty regarding the potential significance of this interface. To quantify the use of retention ponds on commercial poultry farms by wild waterfowl, we surveyed 12 such ponds across Somerset and Dorchester counties, Maryland, USA. This region was chosen due to the high level of poultry production and its importance for migratory waterfowl. Surveys consisted of recording waterfowl visible on the retention ponds from public roadways at least once per week from 20 September 2022–31 March 2023. Throughout the course of this study, we observed a total of nine species of waterfowl using retention ponds on commercial poultry farms at nine of 12 sites. The number of waterfowl observed at retention ponds varied notably throughout the course of our survey period, with values generally following trends of fall migration within each species indicating that resident birds were not the only individuals to utilize these habitats. Additionally, waterfowl use was highest at sites with little vegetation immediately surrounding the pond, and lowest when ponds were surrounded by trees. Our data suggest that retention ponds on commercial poultry farms present a notable interface for waterfowl to introduce avian influenza viruses to farm sites. However, additional testing and surveys could provide further insight into whether it may be possible to reduce the use of these habitats by wild waterfowl through vegetative management as preliminarily reported here.

## 1. Introduction

The recent incursion of highly pathogenic avian influenza (HPAI) into the United States [1, 2] had large ecological and financial ramifications [3]. Wild waterfowl, which are natural reservoirs of low pathogenic avian influenza (LPAI; [4, 5]), have expressed a wide range of responses to infection based on species and location [6–8]. Concurrently, the commercial poultry industry has seen outbreaks spread across the country, with outbreaks at 438 commercial flocks across 32 states and 600 backyard flocks across 47 states as of December 2023 [9]. Cumulatively, more than 78 million domestic poultry have either died directly from infection or as a result of control efforts [9].

Given the impact of HPAI outbreaks on the commercial poultry industry, there has been extensive interest in understanding how avian influenza viruses (AIV) first enter commercial poultry facilities [10, 11]. While poultry grown in backyard or free-range flocks are able to have direct contact with wild birds [12], biosecurity practices at most commercial farms in the United States prevent traditional direct or fecal-oral transmission [13]. Therefore, the specific mechanism(s) allowing initial introduction events [2] remain unclear. One hypothesized method by which AIV could enter commercial poultry facilities involves human-mediated transmission, where personnel entering barns transport virus on their shoes, clothes, or skin or on equipment being brought into the barn [14–16]. Similarly, virus could be entering facilities via contaminated feed or water [14–16]. An additional potential method of viral introduction is via aerosolized virus entering through the barn's ventilation [17], though this has previously been discussed primarily in the context of farm-to-farm transmission [18, 19]. Finally, some researchers suspect that transmission into commercial poultry facilities could occur via bridge species, such as rodents and passerines, which are both capable of contracting and transmitting avian influenza, coming into contact with the virus on the landscape surrounding a farm, and transporting it into the barn themselves [20–22].

While the specific mechanism(s) by which AIV is initially introduced into commercial poultry facilities requires further research, studies have identified several landscape and farm characteristics that elevate AIV transmission risk across the wild waterfowl–domestic poultry interface. For instance, the type of poultry being grown at a given farm appears to play a meaningful role in transmission risk, with turkeys being more susceptible than chickens [23–25]. Similarly, as discussed above, the containment of birds (i.e., allowed outside or kept within buildings) is also a major risk factor [13]. However, the single most important element of transmission risk appears to be the presence of wild waterfowl on the surrounding landscape [26, 27]. The impact of wild waterfowl on the risk of AIV introduction events is demonstrated by a study with blue-winged teal [27] that found that as waterfowl residence time increased so too did the likelihood of an outbreak.

Despite the well-established disease risks associated with wild waterfowl in close proximity to commercial poultry facilities, many poultry farms have retention ponds immediately outside poultry houses [28, 29]. These ponds serve as a means of controlling nutrient runoff and reducing the environmental impact of poultry farming on local waterways [28]. However, these ponds also provide potential habitat for wild waterfowl, as demonstrated by the observation of dabbling ducks on similar waterbodies at a poultry farm in the Netherlands [30]. The risk of attracting waterfowl to the farm property via the presence of a retention pond is further compounded by the ability of AIV to persist in the aqueous environment [31]. Thus, the presence of these retention ponds provides an opportunity not only for viral shedding near the poultry barns but also gives an interface for bridge species to encounter AIV and facilitate entry into the facility [22].

Interestingly, very little is known about the use of retention ponds on commercial poultry farms by wild waterfowl. Given the potential for retention ponds to facilitate the introduction of AIV from wild to domestic birds, improving our understanding of how and when waterfowl use these waterbodies is an important next step in informing appropriate management and risk mitigation practices. This study was a preliminary exploration of the potential for disease transmission by waterfowl in these retention ponds. The objective of this study was to conduct surveys of retention ponds located on commercial poultry farms on the eastern shore of Maryland to determine if waterfowl use these waterbodies. Additionally, we sought to identify any apparent trends in how landscape characteristics impacted the use of individual ponds.

## 2. Methods

This study took place in Dorchester and Somerset counties along the eastern shore of Maryland (portion of Maryland bordering the eastern shore of the Chesapeake Bay), a part of the broader Delmarva Peninsula (an area containing Delaware and the eastern shores of Maryland and Virginia). The Delmarva Peninsula was chosen for this study due to the high level of poultry production in this region. In 2022 alone, ∼596 million chickens were grown on Delmarva, yielding 4.4 billion pounds of chicken [32]. Additionally, the Delmarva region is a key habitat for migratory waterfowl populations along the Atlantic Flyway with many using the Chesapeake Bay as stopover or wintering habitat [33, 34]. This convergence of poultry and wild waterfowl puts the Delmarva Peninsula at elevated risk for AIV transmission across the wild bird–domestic poultry interface [27], with several farms in this region impacted by the ongoing HPAI outbreak [9]. Thus, this region presented an ideal environment to understand the potential local scale risk that may be presented by waterfowl use of retention ponds on poultry farms.

### 2.1. Study Area

To identify retention ponds associated with commercial poultry farms, we manually reviewed each waterbody within a publicly available digitized dataset of all waterbodies within 500 m of commercial poultry facilities on Delmarva in 2016 [35]. We restricted the dataset to just sites within Dorchester and Somerset counties (Maryland) and then retained only those ponds which met the following criteria. First, ponds had to be located on the same physical farm property as the poultry houses (i.e., those in residential backyards were excluded). Second, we only included those waterbodies that appeared to function as a retention pond (i.e., those that were obvious rivers were excluded). Finally, all sites had to be visible from a publicly accessible roadway and appear to be active poultry farms. While conducting observations via game cameras on the farms themselves would have been preferred, such an approach would require regularly moving between multiple farm properties and entering farm premises to access cameras. Thus, the risk of transporting AIV from site to site during the ongoing HPAI outbreak was deemed too high to justify during this initial evaluation. To protect the identities of individual farms, we do not provide the locational coordinates of specific sites.

### 2.2. Survey Methods

Surveys were conducted one to two times per week from 20 September 2022–31 March 2023 with surveys beginning within 1 hr of sunrise. During each survey, we recorded the number of each waterbird species present both within and immediately around (within ∼10 m) the pond as well as the percentage of the pond that was visible to observers. While the focus was on waterbird species, general notes were also taken of other avian species observed. Sites were surveyed in one of two established orders, such that all sites were surveyed in the same order within each of our two counties but the order in which each county was surveyed alternated. As noted above surveys were conducted from the shoulder of publicly accessible roadways to avoid risk of transmitting AIV between sites, with each site surveyed for the length of time needed to count all waterbirds in and around the waterbody (ranging from 1 to 19, $\bar{x}=2.1$ min). All survey data are available in Sullivan et al. [36].

### 2.3. Covariates and Analysis

To assess the role surrounding habitat may play in the use of retention ponds by waterfowl, a photograph of each pond in our study was taken once per week, with an effort made to ensure images were collected at a consistent location, focal depth, and framing. These images were then used to classify each site as brush, open, or tree dominant. Ponds characterized as brush dominant were directly buffered by brush-like vegetation such as cattails (*Typha* spp.) and fully grown little bluestem (*Schizachyrium scoparium*) as well as occasionally more considerable vegetation such as shrubs. Meanwhile, open ponds were buffered by vegetation that was routinely cut and maintained at ground level. Finally, sites were considered tree dominant ponds if they had a dense line of trees directly buffering the pond. While more than one of these categories of vegetation may have been present at a given site, categorizations were based on the dominating feature. Note that our categorizations looked only at the vegetation directly buffering each pond site, without regard to the wider surrounding landscape (i.e., features at the scale of hundreds of meters) which may influence general habitat value but not the ability of waterfowl to access the specific pond observed. Also, while differing vegetation could impact detectability, all study sites were clearly visible and thus we believe it is unlikely that vegetation impacted observations. Finally, we measured the size of each pond and the distance from the pond to the nearest public road within a GIS platform (ArcGIS Pro, Esri, Redlands, California). These values were obtained via the “measure tool” instead of calculating geometry of polygons from the original digitized source [35] due to source polygons often not accurately following pond contours.

**Figure 1:**
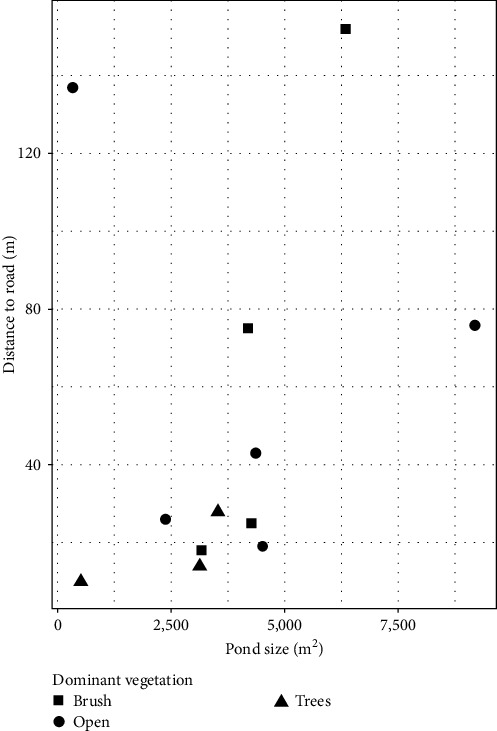
The distribution of retention pond survey sites across major covariates considered in this study (dominant surrounding vegetation, distance to road, and pond size). All data supporting this figure are available from Sullivan et al. [36].

It should be noted that no statistical analyses were performed in this study, with all trends being described qualitatively. This decision was made due to the limited range of sample sites across our identified covariates (Figure 1). When this was paired with potentially confounding effects such as broader surrounding landscape and the short observation windows (see Discussion), we felt presenting statistical results risked implying undue confidence in specific values observed. Instead, we focus on general trends identified from this preliminary evaluation of the use of retention ponds on commercial poultry farms by waterfowl.

## 3. Results

From 20 September 2022–31 March 2023, we surveyed 12 retention ponds associated with commercial poultry facilities on the Delmarva Peninsula. Surveys were conducted across 37 individual days yielding a total of 440 individual site surveys conducted (some sites were not surveyed on all dates due to road restrictions or other access limitations). A total of 10 waterfowl species were observed using the retention ponds (Table 1), with waterfowl observations made at nine of our 12 sites. Canada geese (*Branta canadensis*) were the most common with observations occurring at six different retention ponds including a maximum of 132 individuals seen during a single observation period. In addition to waterfowl, numerous additional waterbirds were seen at our survey sites including Great Blue Herons (*Ardea herodias*) and Bald Eagles (*Haliaeetus leucocephalus*).

**Figure 2:**
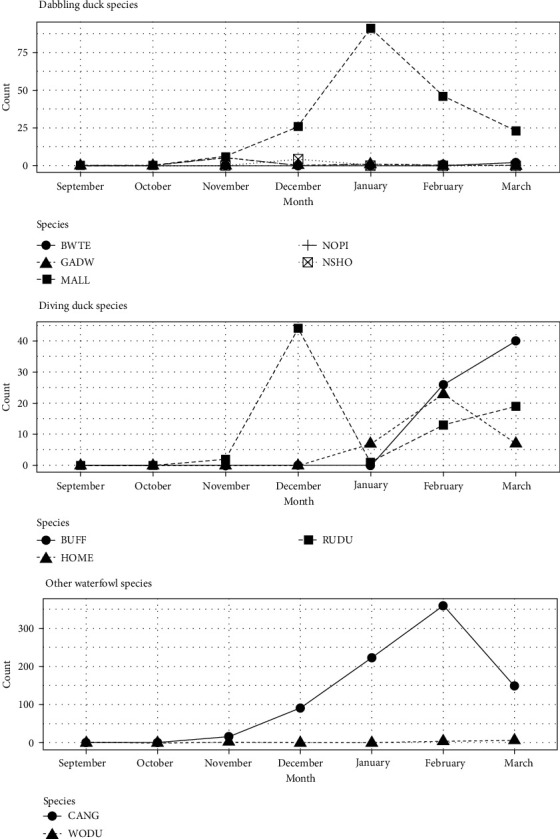
The number of waterfowl, by species, observed on retention ponds associated with commercial poultry facilities in Dorchester and Somerset counties, Maryland, from 20 September 2022 to 31 March 2023. Species are identified via the standard four-letter alpha codes (BWTE, blue-winged teal; GADW, gadwall; MALL, mallard; NOPI, northern pintail; NSHO, northern shoveler; BUFF, bufflehead; HOME, hooded merganser; RUDU, ruddy duck; CANG, Canada goose; and WODU, wood duck). All data supporting this figure are available from Sullivan et al. [36].

The number of waterfowl observed at retention ponds varied notably throughout the course of our survey period. For instance, mallard observations appeared to follow the fall migratory trends, with sightings increasing in December before a peak in January and subsequent decline in February (Figure 2). While Canada geese followed this same general trend of increasing during fall migration, peak encounters did not occur until February. Temporal patterns were variable among diving duck species, with peak observation ranging from December for Ruddy Ducks (*Oxyura jamaicensis*) to March for Buffleheads (*Bucephala albeola*).

**Figure 3:**
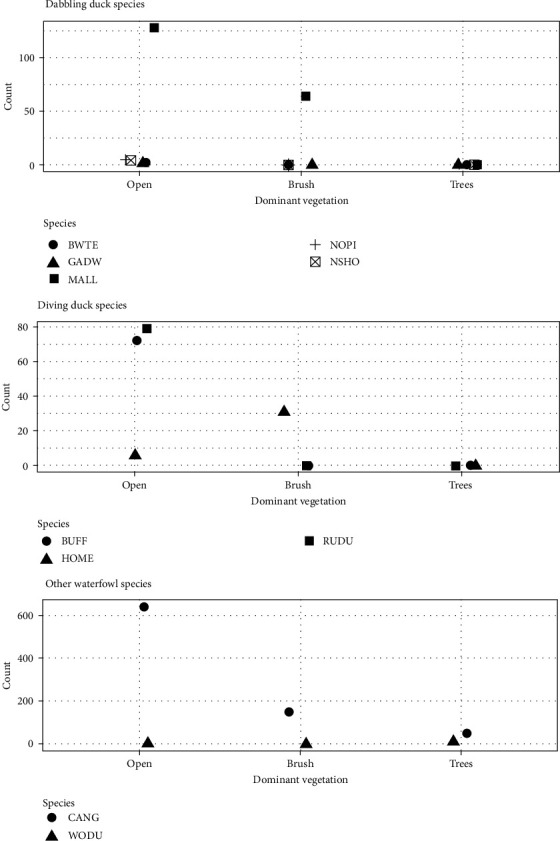
The impact of vegetation immediately surrounding retention ponds on commercial poultry farms on the number of waterfowl observed utilizing the ponds. Species are identified via the standard four-letter alpha codes (BWTE, blue-winged teal; GADW, gadwall; MALL, mallard; NOPI, northern pintail; NSHO, northern shoveler; BUFF, bufflehead; HOME, hooded merganser; RUDU, ruddy duck; CANG, Canada goose; and WODU, wood duck). All data supporting this figure are available from Sullivan et al. [36].

**Figure 4:**
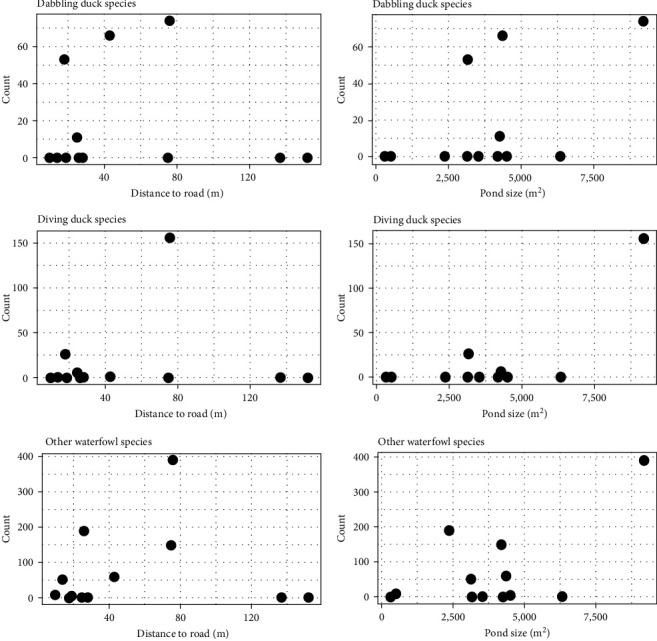
The impact of distance to nearest road and pond size on the number of waterfowl observed utilizing retention ponds associated with commercial poultry farms. Species are broken into three groups: dabbling ducks (blue-winged teal, gadwall, mallard, northern pintail, northern shoveler), diving ducks (bufflehead, hooded merganser, and ruddy duck), and other waterfowl (Canada goose and wood duck). All data supporting this figure are available from Sullivan et al. [36].

The vegetation immediately surrounding each site also appeared to impact waterfowl use of retention ponds. Ponds exhibiting open vegetation had the most species as well as the highest counts within individual species (Figure 3). Conversely, the lowest number of species were observed at tree dominated ponds, where only wood ducks (*Aix sponsa*) and Canada geese were observed. However, the observations at tree dominated ponds were confounded by notable issues. For instance, there was one tree dominated site at which no waterfowl were documented, but the landowner indicated that he actively harasses wild birds to minimize disease transmission risk. Conversely, the two tree dominated ponds where waterfowl were observed both had a wood duck nesting box along the ponds' edge. Finally, the impact of pond size and distance to road appeared to be highly variable (Figure 4).

## 4. Discussion

Our data indicate that retention ponds on commercial poultry farms along the eastern shore of Maryland are being utilized by wild waterfowl and, therefore, serve as a potential interface for the transmission of avian influenza. Indeed, the variety of waterfowl species observed, and the large number of sites used (75% of study sites) suggests that the use of these retention ponds by waterfowl is neither rare nor inconsequential. These results are especially noteworthy given the limited survey effort at each site, which would suggest that our results show a very conservative estimate of waterfowl presence at these locations with the actual species composition likely being higher and more frequent than captured in our results.

While the diversity and volume of waterfowl observed using retention ponds associated with commercial poultry facilities was a surprising result, the temporal trends in usage aligned well with the abundance of these species in this region [37, 38]. For instance, peak numbers of mallards and Canada geese on retention ponds were observed in December and January, respectively, while peak numbers of later migrants such as buffleheads were observed in March. Importantly, the increase in observed use of retention ponds by waterfowl corresponding to migratory pulses in local abundance suggests that the use of these habitats is not dominated by resident birds. This has important implications for AIV transmission, as migratory waterfowl are believed to play the largest role in viral dissemination along a flyway [33, 39, 40]. It should also be noted that this period of fall migration corresponds with increased AIV prevalence in the species observed in this study [41, 42], presenting an elevated threat to the poultry operations associated with retention ponds utilized by these waterfowl compared to if use occurred in a period such as May through June when viral prevalence is low [42].

Another important factor in the use of retention ponds near commercial poultry operations by waterfowl appears to be the vegetation immediately surrounding the pond. Our data align with previous research that suggests waterbodies buffered by dense stands of tall, emergent vegetation demonstrate decreased use by waterfowl, whereas waterbodies that are sparsely vegetated with shorter emergent vegetation in a more open wetland habitat depict increased utilization by wild waterfowl [43]. For instance, Canada geese have been found to seek waterbodies with an open shoreline possessing good visibility, as they need a direct line of sight to see predators approaching and require additional time to become airborne and evade predators due to their size and weight [44]. This trend was also found at our retention ponds, with notable declines in cumulative observations of Canada geese as vegetation intensified. The only species that was seen in higher numbers at tree dominated sites versus open sites were wood ducks, which fits the breeding ecology of this species [45].

While it would likely be ill-advised to remove retention ponds from poultry farm complexes due to nutrient management needs [28], our data suggest that managing vegetation surrounding these ponds could reduce their usage by wild waterfowl, and thus mitigate some amount of AIV transmission risk. For instance, a dense border of coniferous trees may be suitable to prevent use by some waterfowl species [46]. Still, as this hypothesis was not directly tested in this study, additional research is needed on the efficacy of this approach. Similarly, other pond management approaches such as altering pond depth or controlling aquatic vegetation may impact use by migratory waterfowl [47], though these factors were also not evaluated in this study.

Despite the valuable trends identified in this study, there are several limitations that should be considered when interpreting results. First, this study represents short observations from a limited number of sites collected within only certain seasons of a single year. Similarly, this effort did not account for habitat composition at the larger landscape level which could also impact use of these local scale sites [48]. To allow for analyses that fully parse out the role of all pertinent factors and appropriately conclude if there are habitat management approaches that could meaningfully reduce waterfowl presence at this transmission interface additional research is still required. Such efforts would likely benefit from a direct collaboration between researchers and commercial poultry producers that would allow for camera traps to be placed directly on farm property overlooking the retention ponds. Such an approach would allow for a greater sample size and monitoring frequency than was achievable in this human-observer-based approach [49]. By having a remote monitoring approach additional biases in our study, such as diurnal only data collection, could be removed. It is well-established that some waterfowl species forage in the evening hours, so our study may be overly conservative regarding use of the retention pond habitats [30]. Additionally, being able to access ponds not visible from public roadways would allow inclusion of a wider range of values for factors such as distance from road and pond size.

## 5. Conclusions

The data presented here demonstrate that a wide-ranging assemblage of waterfowl are utilizing retention ponds associated with commercial poultry farms on the eastern shore of Maryland, with increasing use observed during fall migration. While these retention ponds serve an important role in nutrient management, attracting waterfowl to poultry farms also presents an inherent risk for the transmission of avian influenza. While this study alone does not provide an actionable assessment of factors that impact likelihood of retention pond use by waterfowl, due to limited scale and geographic scope, it does identify promising areas for risk abatement such as managing vegetation around retention ponds which could be studied further.

## Figures and Tables

**Table 1:** A summary of waterfowl observed on retention ponds associated with commercial poultry farms in Dorchester and Somerset counties, Maryland, from 20 September 2022 to 31 March 2023.

Species	Sites observed	Max observed
Blue-winged teal	2	2
Bufflehead	2	14
Canada goose	6	132
Gadwall	1	1
Hooded merganser	3	12
Mallard	4	16
Northern pintail	1	5
Northern shoveler	2	3
Ruddy duck	1	20
Wood duck	3	2

Values indicate the number of sites at which a given species was observed and the maximum number of individuals from that species observed during a single survey. All data supporting this figure are available from Sullivan et al. [36].

## Data Availability

All data supporting the results of this manuscript are provided as supplemental material and are published openly (Sullivan et al. [36]; https://doi.org/10.5066/P9U7QISZ).

## Supplementary Materials

Supplementary Material The file “Suuplemental_Data.txt” contains all data generated in this study and supports all analyses reported. These data are also available from Sullivan et al. [36] (https://doi.org/10.5066/P9U7QISZ).
